# Kinetics of N‐ to M‐Polar Switching in Ferroelectric Al_1−x_Sc_x_N Capacitors

**DOI:** 10.1002/advs.202308797

**Published:** 2024-02-14

**Authors:** Roberto Guido, Haidong Lu, Patrick D. Lomenzo, Thomas Mikolajick, Alexei Gruverman, Uwe Schroeder

**Affiliations:** ^1^ NaMLab gGmbH Noethnizer Strasse 64a 01187 Dresden Germany; ^2^ Chair of Nanoelectronics Technische Universität Dresden Noethnizer Strasse 64 01187 Dresden Germany; ^3^ Department of Physics and Astronomy University of Nebraska Lincoln NE 68588 USA

**Keywords:** aluminum scandium nitride, domains, ferroelectricity, piezoresponse force microscopy, switching kinetics

## Abstract

Ferroelectric wurtzite‐type aluminum scandium nitride (Al_1−x_Sc_x_N) presents unique properties that can enhance the performance of non‐volatile memory technologies. The realization of the full potential of Al_1−x_Sc_x_N requires a comprehensive understanding of the mechanism of polarization reversal and domain structure dynamics involved in the ferroelectric switching process. In this work, transient current integration measurements performed by a pulse switching method are combined with domain imaging by piezoresponse force microscopy (PFM) to investigate the kinetics of domain nucleation and wall motion during polarization reversal in Al_0.85_Sc_0.15_N capacitors. In the studied electric field range (from 4.4 to 5.6 MV cm^−1^), ferroelectric switching proceeds via domain nucleation and wall movement. The currently available phenomenological models are shown to not fully capture all the details of the complex dynamics of polarization reversal in Al_0.85_Sc_0.15_N. PFM reveals a non‐linear increase of both domain nucleation rate and lateral wall velocity during the switching process, as well as the dependency of the domain pattern on the polarization reversal direction. A continuously faster N‐ to M‐polar switching upon cycling is reported and ascribed to an increasing number of M‐polar nucleation sites and density of domain walls.

## Graphical abstract

The domain structure dynamics during nitrogen‐ to metal‐polar switching in ferroelectric aluminum scandium nitride capacitors are investigated through transient current integration measurements and piezoresponse force microscopy. The ferroelectric switching process relies on the complex interplay between domain nucleation and wall motion. The relative contribution of domain nucleation and wall motion to the polarization reversal varies during the switching process and upon cycling.

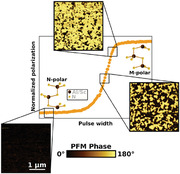

## 1 Introduction

The growing demand for low‐power, non‐volatile, and high‐density memories is driving an intensive exploration of alternatives to charge‐based storage technologies, which could eventually lead to bridging the gap between storage and computation.^[^ ^1^, ^2^, ^3^, ^4^, ^5^ ^]^ The field‐driven switching mechanism combined with stable and long retention of the remanent polarization (P_r_) makes ferroelectrics an ideal candidate for realizing the next generation of non‐volatile memory technologies.^[^ ^2^, ^5^, ^6^, ^7^ ^]^

The first reports of ferroelectricity in wurtzite‐type (space group *P*6_3_ *mc*) aluminum scandium nitride (Al_1−x_Sc_x_N) created attention because of unique material properties that combined with a demonstrated scalability below 5 nm and complementary metal oxide semiconductor back‐end‐of‐line compatibility can enhance the performance of ferroelectric devices.^[^ ^8^, ^9^, ^10^, ^11^ ^]^ In particular, Al_1−x_Sc_x_N is an ideal fit for the integration of ferroelectrics into gallium nitride‐based high electron mobility transistors.^[^ ^12^, ^13^ ^]^ The successful integration of Al_1−x_Sc_x_N in the gate stack of field‐effect transistors with two‐dimensional channel materials, and into analog synaptic memristors was also demonstrated.^[^ ^9^, ^14^, ^15^ ^]^ Nonetheless, realizing the full potential of Al_1−x_Sc_x_N requires a comprehensive understanding of the mechanism of polarization reversal and domain structure dynamics involved in the ferroelectric switching process. Scanning transmission electron microscopy (STEM) analyses combined with first‐principles calculation predictions showed that in AlN‐based ferroelectrics, the polarization reversal is achieved through the complete and homogeneous inversion of the wurtzite‐type structure between a nitrogen (N)‐polar state and a metal (M)‐polar state mediated through various intermediate states including a metastable transient non‐polar structure.^[^ ^16^, ^17^ ^]^ Several authors reported on the temperature dependence of the electric field required for reversing the polarization orientation in wurtzite‐type ferroelectrics, proposing that the switching proceeds via domain nucleation and growth rather than an intrinsic process, as instead described in the Landau–Ginzburg theory.^[^ ^18^, ^19^, ^20^, ^21^ ^]^ Recently, STEM allowed observation of the ferroelectric domains within an Al_1−x_Sc_x_N grain.^[^ ^10^ ^]^ Ferroelectric Al_1−x_Sc_x_N was shown to have a narrower local electric field distribution compared to lead zirconate titanate (PZT) and doped hafnia, leading to abrupt switching when large electric field magnitudes are applied.^[^ ^22^, ^23^ ^]^ However, no conclusive evidence of how the domain structure evolves during ferroelectric switching in Al_1−x_Sc_x_N has emerged so far. Mapping the domain structure dynamics during N‐ to M‐polar switching down to the nanoscale is required to improve the reliability of ferroelectric devices, as well as to develop multilevel memories and artificial synapses for neuromorphic computing.^[^ ^24^, ^25^, ^26^ ^]^

Piezoresponse force microscopy (PFM) enables non‐destructive visualization, control, and measurement of local physical characteristics of ferroelectrics.^[^ ^27^ ^]^ In this work, transient current integration measurements performed by a pulse switching method are combined with domain imaging by PFM to investigate the kinetics of domain nucleation and wall motion during polarization reversal in Al_0.85_Sc_0.15_N capacitors. The Kolmogorov–Avrami–Ishibashi (KAI), nucleation‐limited‐switching (NLS) and simultaneous non‐linear nucleation and growth (SNNG) models are compared to understand which best fits the experimental data in the studied electric field range (from 4.4 to 5.6 MV cm^−1^). Stroboscopic PFM is applied to resolve and follow the evolution of the domain structure dynamics during polarization reversal.^[^ ^27^ ^]^ The completeness and limitations of the SNNG model in describing the Al_0.85_Sc_0.15_N switching kinetics are discussed, considering quantitative data related to domain switching extracted via PFM. Electric field‐dependent visualization of the domain configurations developing in the ferroelectric capacitors and transient current integration measurements are used to investigate the N‐ to M‐polar switching process upon cycling.

## 2 Results and Discussion

**Figure 1:**
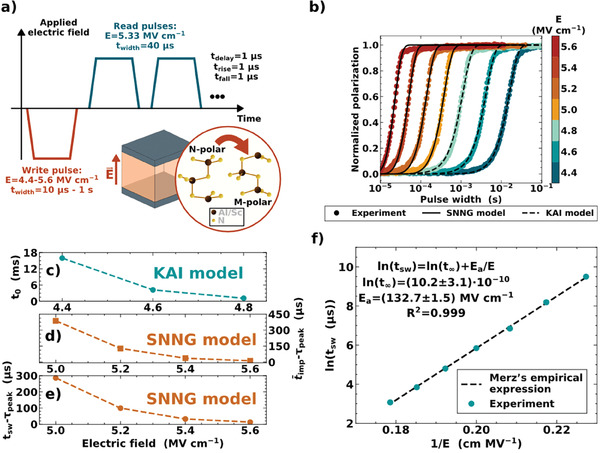
a) Pulse sequence applied to the capacitors for the transient current integration measurements. For a given electric field magnitude (E) during the write pulse, the entire pulse sequence is repeated with a varying write pulse width (t_width_) from 10 µs to 1 s. The rise (t_rise_) and falling (t_fall_) times of each pulse, as well as the delay time between the pulses (t_delay_), are indicated in the figure. A simplified sketch of how the Al_0.85_Sc_0.15_N structure changes after the N‐ to M‐polar ferroelectric switching is shown close to the write pulse. b) Normalized polarization extracted from transient current integration measurements vs. pulse width for each applied electric field magnitude (E) between 4.4 and 5.6 MV cm^−1^. The best fitting by the SNNG or KAI model is shown. c) Characteristic switching time (t_0_) for the KAI model vs. applied electric field magnitude. d) Difference between the average impingement time (${\bar{t}}_{\mathrm{imp}}$) and the time at which the non‐linear increase of the nucleation rate peaks (τ_peak_) for the SNNG model vs. applied electric field magnitude. e) Difference between the switching time (t_sw_) and the time at which the non‐linear increase of the nucleation rate peaks (τ_peak_) for the SNNG model vs. applied electric field magnitude. f) Natural logarithm of the switching time (t_sw_) vs. inverse of the applied electric field magnitude (1/E). The data points are fit using Merz's empirical expression. The fitting parameters and the coefficient of determination (R^2^) of the fit are reported.

The kinetics of ferroelectric switching was investigated in a highly textured 60 nm thick Al_0.85_Sc_0.15_N film sandwiched between a titanium nitride (TiN) top electrode (TE) and a titanium/tungsten (Ti/W) bottom electrode (BE) using transient current integration measurements with the pulse sequence shown in **Figure** **1**a. As it is demonstrated later by PFM, the ferroelectric film is homogeneously polarized in the N‐polar state (the positive end of the ferroelectric dipole points to the substrate) right after deposition. An initial write pulse resulting in an electric field directed toward the TE is applied to gradually switch the ferroelectric film to the M‐polar state. Subsequently, two read pulses having opposite polarity compared to the write pulse are applied to extract the amount of switched polarization while compensating for the capacitive displacement and leakage current components. For a given electric field magnitude, the time‐dependent polarization evolution from N‐ to M‐polar state can be obtained by varying the write pulse width, as shown in Figure 1b.

The polarization reversal process consists of the combination of domain nucleation and wall motion. Three main phenomenological scenarios can describe the switching process. Ferroelectric switching can be governed by the growth of reversed domains through wall motion until impingement, as described through the KAI model, or by the statistics of independently reverse nucleating domains, as referred to as the NLS model.^[^ ^28^, ^29^ ^]^ Recently, the SNNG model, which introduces a non‐linear increase of the nucleation rate occurring simultaneously with domain growth, was proposed to extend the KAI model and describe the switching kinetics in wurtzite‐type ferroelectrics under large electric field magnitudes.^[^ ^23^ ^]^ A more thorough overview of these three phenomenological models is presented in Section S1 (Supporting Information).

These three main phenomenological theories were compared to find which best describes the switching kinetics in Al_0.85_Sc_0.15_N (see also Section S2, Supporting Information). Under the application of electric field magnitudes below 5.0 MV cm^−1^, the experimental data shown in Figure 1b are adequately represented by the KAI model alone without introducing more complicated models, such as the SNNG. Increasing the applied electric field magnitude from 4.4 to 4.8 MV cm^−1^ results in a faster domain wall motion and, hence, in a lower characteristic switching time (t_0_), as shown in Figure 1c.^[^ ^28^ ^]^ Nonetheless, for applied electric field magnitudes above 5.0 MV cm^−1^, a discrepancy between the experimental data and the KAI model emerges, and the SNNG model provides a better fit of the switching kinetics data. The crossover between the KAI and the SNNG models is due to the non‐negligible contribution of the non‐linear increase of the nucleation rate, which is not accounted for in the KAI model.^[^ ^23^ ^]^ Comparing this result with the study of Yazawa et al.^[^ ^23^ ^]^ shows that macroscopically the kinetics of ferroelectric switching in Al_1−x_Sc_x_N is not affected by scaling the film thickness down to 60 nm. Increasing the applied electric field magnitude from 5.0 to 5.6 MV cm^−1^, the time at which the non‐linear increase of the nucleation rate peaks (τ_peak_) gets closer to the switching time (t_sw_), as reported in Figure 1e. Here, t_sw_ is defined as the time at which half of the polarization is reversed from N‐ to M‐polar. By considering the average impingement time (${\bar{t}}_{\mathrm{imp}}$) as the average time required for the coalescence of two growing domains, Figure 1d shows that increasing the applied electric field magnitude causes a reduction in the difference between ${\bar{t}}_{\mathrm{imp}}$ and τ_peak_. Thus, nucleation events and domain wall motion of already reverse nucleated domains become closer in time with larger magnitudes of the applied electric field until the expected simultaneous occurrence of the two phenomena. In this regard, it is worth mentioning that for all the investigated electric field magnitudes, significant domain growth always follows the peak in nucleation rate because ${\bar{t}}_{\mathrm{imp}}$ is larger than τ_peak_. This observation highlights a surprising difference in switching kinetics between wurtzite‐type Al_1−x_Sc_x_N and other ferroelectric thin films, such as polycrystalline PZT or doped hafnia, in which the NLS model usually describes the mechanism of polarization reversal at room temperature.^[^ ^29^, ^30^, ^31^ ^]^ The significant domain wall motion contribution to the ferroelectric switching kinetics could hinder the need for extremely large energy to nucleate domains of opposite polarity, which implies a low density of pre‐existing nuclei. It would be more energetically favorable to expand the already nucleated domains to achieve the full polarization reversal compared to the occurrence of additional isolated nucleation events.^[^ ^32^ ^]^ The t_sw_ follows Merz's empirical expression (Figure 1f).^[^ ^33^, ^34^ ^]^ The activation field E_a_ for ferroelectric switching extracted from Figure 1f is found to be (132.7 ± 1.5) MV cm^−1^. Coherently with the magnitude of the E_c_, the E_a_ in Al_0.85_Sc_0.15_N is larger than that reported for PZT and doped hafnia by three and two orders of magnitude respectively.^[^ ^35^, ^36^ ^]^ The extracted E_a_ is of the same order of magnitude but larger than the value computed using the inhomogeneous field distribution model by Do Kim et al.^[^ ^22^ ^]^ for Al_0.7_Sc_0.3_N, which is consistent with the larger E_c_ in the studied Al_0.85_Sc_0.15_N due to the lower scandium content. Merz associated the E_a_ to the activation for the nucleation of oppositely poled domains, thus the large E_a_ agrees with the hypotheses of the large energy required for nucleation and low density of pre‐existing nuclei of opposite polarity in the pristine Al_0.85_Sc_0.15_N film. Nonetheless, the lateral domain wall motion plays a decisive role in the dynamics of ferroelectric switching in Al_0.85_Sc_0.15_N, in contrast to the barium titanate system studied by Merz. Hence, the large E_a_ magnitude for Al_0.85_Sc_0.15_N could account for the slow sideways domain wall motion related to domain wall pinning‐depinning transitions. In this regard, the maximum depolarization field in the absence of screening charges is similar to the E_a_, which corresponds to the threshold for the transition of domain wall motion from creep regime to flow regime (see Section S3, Supporting Information).^[^ ^37^ ^]^

**Figure 2:**
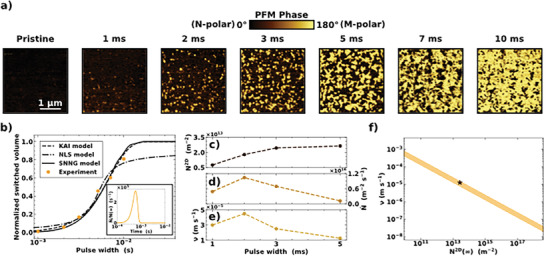
a) PFM phase images (3 × 3 µm) of the time‐dependent switching behavior when applying a 5.0 MV cm^−1^ electric field directed toward the TE of an initially pristine capacitor. The scale bar of 1 µm applies to all PFM phase images. b) Fitting of the normalized volume fraction of switched domains extracted from the PFM phase images vs. pulse width by KAI, NLS, and SNNG models. The inset shows the normalized non‐linear nucleation rate distribution for the SNNG model. c) Two‐dimensional nucleation density (N^2D^) extracted from the PFM phase images vs. pulse width. d) Nucleation rate ($\dot{N}$) extracted from the PFM phase images vs. pulse width. e) Domain wall velocity (ν) extracted from PFM phase images vs. pulse width. f) Domain wall velocity (ν) vs. saturated two‐dimensional nucleation density (N^2D^(∞))). The shaded region indicates the confidence interval considering three standard deviations of the fitting parameters of the SNNG model. The star symbol refers to the ν extracted from the time‐dependent PFM phase images when N^2D^(∞)is reached. The N^2D^(∞)axis is limited to a range from one nucleus in the measured capacitor to one nucleus per unit cell.

The fitting of the integrated transient current data shows that both domain nucleation and wall motion play a role in the switching kinetics of wurtzite‐type Al_1−x_Sc_x_N capacitors. However, limiting the investigation to macroscopic measurements and the currently available phenomenological descriptions (KAI, NLS, and SNNG models) of the ferroelectric switching process may hinder important details of this extrinsic mechanism. To capture and follow the evolution of the domain configurations developing in ferroelectric capacitors during polarization reversal, stroboscopic PFM was applied.^[^ ^35^ ^]^ From the time‐dependent PFM phase images in **Figure** **2**a it can be observed that the pristine Al_0.85_Sc_0.15_N capacitor is in the homogeneous N‐polarity and that ferroelectric switching proceeds through three main steps: i) nucleation of M‐polar domains; ii) sideways expansion of already nucleated M‐polar domains and simultaneous occurrence of additional nucleation events; iii) domain growth by lateral wall motion accompanied by a negligible contribution of nucleation. The two‐dimensional domain growth observable in the PFM phase images reported in Figure 2a implies a much faster domain growth through the film thickness compared to the sideways expansion. The irregular shape of the growing domains indicates their interaction with structural defects during sideways domain expansion.^[^ ^36^, ^38^ ^]^ By extracting the volume fraction of switched domains versus the write pulse width for the applied electric field magnitude of 5.0 MV cm^−1^, the domain switching kinetics can be equally well described mathematically by all three phenomenological models using the set of data in Figure 2b. Additional data points for longer pulse widths than 10 ms would be required to conclude which of the three phenomenological models better describes the experimental data shown in Figure 2b. For the capacitors measured in this work, the electrical breakdown prevented the acquisition of the PFM phase images when write pulses longer than 10 ms were applied. Nonetheless, the absence of an increase in the half‐width at the half‐maximum of the Lorentzian distribution of the local switching times for the NLS model when the applied electric field magnitude during the stroboscopic PFM experiment is decreased from 5.0 to 4.5 MV cm^−1^ supports the explanation of the switching kinetics in Al_0.85_Sc_0.15_N capacitors provided by KAI and SNNG models (see Section S4, Supporting Information). Quantitative data regarding the dynamics of the switching process in terms of two‐dimensional nucleation density (N^2D^), nucleation rate ($\dot{N}$) and lateral wall velocity (ν) were extracted from the time‐dependent PFM phase images, as reported in Figure 2c,d,e. The two‐dimensional nucleation density continuously increases with pulse width saturating when ≈81% of the capacitor volume is switched. In agreement with the SNNG model, the nucleation rate follows a non‐linear increase. The drop of the nucleation rate by almost one order of magnitude, when the pulse width is 5 ms, suggests the transition from a nucleation‐limited regime to a predominantly sideways domain wall motion regime.^[^ ^36^ ^]^ The maximum nucleation rate is four orders of magnitude lower compared to PZT and doped hafnia ferroelectrics.^[^ ^35^, ^36^ ^]^ This result would agree with the reported larger E_a_ for the nucleation of domains with opposite polarity in the Al_0.85_Sc_0.15_N film studied in this work compared to PZT and doped hafnia ferroelectrics. Nonetheless, the non‐linear increase of nucleation rate extracted from the time‐dependent PFM phase images (Figure 2d) peaks significantly after the normalized nucleation rate extracted from the SNNG fit of the same data (inset of Figure 2b). Recording the stroboscopic PFM phase images through the TE limits the spatial resolution for domain imaging.^[^ ^36^ ^]^ Thus, it could be that the nucleation of M‐polar domains is detected only when they overcome the size close to the resolution limit; hence, the peak in nucleation rate occurs before 2 ms. Another possibility is that the apparent discrepancy between the fit using the SNNG model and the nucleation rate directly extracted from stroboscopic PFM measurement data arises from the phenomenological nature of the SNNG, which does not allow to fully capture all the details of the complex dynamics of polarization reversal in Al_0.85_Sc_0.15_N. The SNNG model assumes a constant value for the lateral domain wall velocity during the switching process.^[^ ^23^ ^]^ On the other hand, a non‐linear increase is encountered when extracting the lateral domain wall velocity from the space‐time dependence of the domain expansion (Figure 2e). The variation of lateral domain wall velocity throughout the switching process could suggest a strong interaction with structural defects, such as grain boundaries.^[^ ^31^ ^]^ This explanation would agree with the larger size of the growing domains compared to the average grain size (≈21 nm), thus a growing domain would encounter several grain boundaries during its sideways expansion. By extracting from the data in Figure 2c,e the saturated value of two‐dimensional nucleation density (N^2D^(∞)) and lateral domain wall velocity when the saturation is reached, a perfect agreement with the SNNG model fit in Figure 2b is obtained (Figure 2f). However, the maximum value of the extracted lateral domain wall velocity is lower than the value reported for PZT or doped hafnia by six and four (or five) orders of magnitude respectively.^[^ ^31^, ^35^, ^36^, ^39^ ^]^ The low lateral domain wall velocity is coherent with the orders of magnitudes lower irreversible Rayleigh coefficient for wurtzite‐type AlN‐based ferroelectrics compared to PZT or doped hafnia at room temperature.^[^ ^40^, ^41^, ^42^ ^]^ On the other hand, in ferroelectrics, the domain wall velocity can follow a non‐linear increase with the applied electric field magnitude.^[^ ^35^, ^38^ ^]^ Thus, the low value of lateral domain wall velocity has also to be ascribed to the applied electric field to measured E_c_ ratio of ≈0.8, and it is expected to get larger by increasing the applied electric field magnitude (see Section S2, Supporting Information). The reference E_c_ magnitude was extracted from the transient current measurement shown in Section S5 (Supporting Information). The small applied electric field to measured E_c_ ratio contributes also to explain the low values of domain nucleation rate in Figure 2d, since the nucleation rate can depend exponentially on the applied electric field magnitude.^[^ ^35^ ^]^

It is worth mentioning that both domain nucleation rate and lateral wall velocity peak at the same pulse width, meaning that there is an acceleration in lateral domain wall velocity with the faster nucleation of oppositely poled domains. This observation could suggest that domains are not independent of each other and that the more oppositely poled domains nucleate, the faster they tend to expand laterally until impingement with neighboring domains.

Comparing the fit of the experimental data acquired by transient current integration measurements and stroboscopic PFM at an applied electric field magnitude of 5.0 MV cm^−1^, ferroelectric switching from N‐ to M‐polar in the wurtzite‐type Al_0.85_Sc_0.15_N film studied in this work can be described using the SNNG model, while considering some model limitations emerging from the PFM results. However, considering the same magnitude of the applied electric field, the polarization reversal is faster during transient current integration measurements (Figure 1b) compared to the time‐dependent PFM experiment (Figure 2b). This unexpected result is ascribed primarily to polarization relaxation and/or depolarization effects, area‐dependent effects, as well as to the influence of the pulse sequence characteristics, such as the width and number of the applied pulses. A more extensive discussion is reported in Section S6 (Supporting Information).

**Figure 3:**
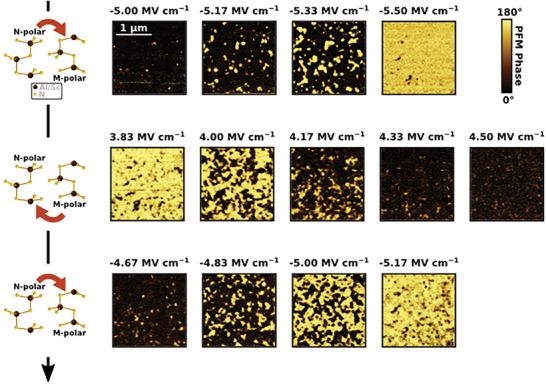
PFM phase images (2 × 2 µm) of the electric field‐dependent switching behavior. Starting from a pristine capacitor, an incrementally increasing negative voltage bias is first applied to the TE causing N‐ to M‐polar switching. The capacitor is switched back to the N‐polar state by applying an incrementally increasing positive voltage bias to the TE. Finally, an incrementally increasing negative voltage bias is applied to the TE to reverse the ferroelectric polarization in the M‐polar state. The corresponding electric field magnitude for the applied voltage bias pulses is reported. The scale bar of 1 µm applies to all PFM phase images.

The electric field‐dependent evolution of the domain structure in the ferroelectric capacitors was recorded by acquiring the PFM phase images after applying voltage pulses with a fixed width but varying amplitude and sign to increase the magnitude and reverse the direction of the electric field applied to the capacitor (**Figure** **3**). Similar to the time‐dependent PFM results, under increasing electric field magnitudes, the mechanism of polarization reversal consists of nucleation of oppositely poled domains and growth for both N‐ to M‐polar transition and vice versa. The smaller electric field magnitude required to switch from M‐ to N‐polar compared to the opposite ferroelectric transition was already reported in previous work, and it can be attributed to the intrinsic larger stability of the N‐ over the M‐polar state and/or to the presence of an internal bias field.^[^ ^26^ ^]^ Figure 3 points out that the nucleation events occur in different regions according to which polarity between N‐ and M‐polar is nucleating, causing the domain pattern to depend on the polarization reversal direction. This result could be explained considering the influence of the defect distribution on the local electric field profile and/or that nucleation occurs at different sites or even at a different interface, according to the polarization reversal direction. Thus, both defect distribution and interface phenomena are suspected to play a decisive role in defining the energy required for ferroelectric switching in wurtzite‐type systems.^[^ ^26^, ^37^, ^43^, ^44^ ^]^

**Figure 4:**
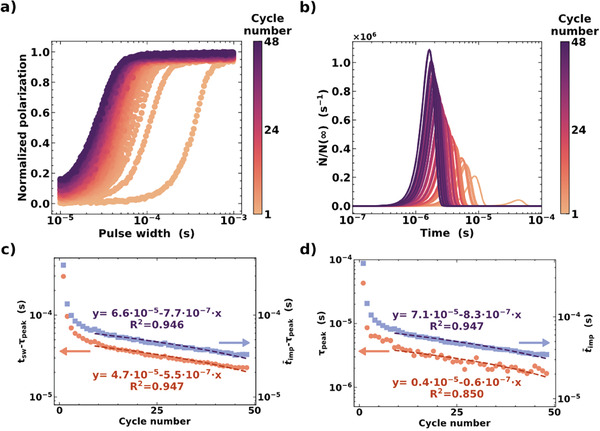
a) Normalized polarization extracted from transient current integration measurements vs. pulse width for consecutive measurement sequences (cycles). The applied electric field magnitude was fixed to 5.0 MV cm^−1^. b) Normalized non‐linear nucleation rate ($\dot{N}/N\left(\infty \right)$) extracted from the SNNG model fit vs. time for consecutive measurement sequences (cycles). c) Difference between the average impingement time (${\bar{t}}_{\mathrm{imp}}$) or switching time (t_sw_) and the time at which the non‐linear increase of the nucleation rate peaks (τ_peak_) vs. measurement sequence (cycle) number. d) Average impingement time (${\bar{t}}_{\mathrm{imp}}$) and time at which the non‐linear increase of the nucleation rate peaks (τ_peak_) vs. measurement sequence (cycle) number. The dashed lines represent the fitting functions. The expression of the fitting function and the coefficient of determination (R^2^) are reported.

By comparing the electric field magnitude at which the pristine to M‐polar and subsequent N‐ to M‐polar switching are achieved in Figure 3, it can be observed that the full N‐ to M‐polar state reversal is attained with ≈0.3 MV cm^−1^ smaller electric field magnitude in the second switching transition. The decrease of the energy barrier for polarization reversal between the N‐ and M‐polar states raises significant reliability concerns for both non‐volatile memory applications and precise weight update in neuromorphic computing architectures.^[^ ^24^, ^26^ ^]^ To investigate the reason for the decrease in electric field magnitude required to switch from the N‐ to the M‐polar state, transient current integration measurements at a fixed electric field magnitude of 5.0 MV cm^−1^ for the write pulse were performed continuously on the same initially pristine ferroelectric capacitor until electrical breakdown. The normalized polarization data for all the transient current integration measurement cycles is reported in **Figure** **4**a. In agreement with previous work, the N‐ to M‐polar switching gets continuously faster while cycling.^[^ ^26^ ^]^ By comparing the result in Figure 4a with Figure 1b, the continuous shift of the normalized polarization curve toward smaller pulse widths would correspond to a reduction of the measured E_c_ by ≈0.6 MV cm^−1^. In wurtzite‐type Al_1−x_Sc_x_N an initial internal bias field is expected due to charge injection that contributes to stabilizing the large P_r_ magnitude in pristine capacitors.^[^ ^26^, ^45^ ^]^ Nonetheless, previous work showed that the internal bias field in the present capacitor stack could not account for the whole reduction in measured E_c_.^[^ ^26^ ^]^ The continuously faster N‐ to M‐polar switching could be explained by the alteration of the local electric field profile due to defect generation and redistribution.^[^ ^26^ ^]^ The defect distribution could also influence the number of nucleation sites and density of domain walls, which are considered responsible for the origin of wake‐up phenomenon in aluminum boron nitride (Al_1−x_B_x_N).^[^ ^23^, ^40^, ^41^ ^]^ In agreement with this hypothesis, Figure 3 shows that the first M‐polar domains nucleate at different regions between the two consecutive N‐ to M‐polar switching transitions. Nonetheless, the Al_0.85_Sc_0.15_N ferroelectric capacitors in this study do not show a clear wake‐up behavior like in the Al_1−x_B_x_N case because almost the full P_r_ magnitude is reached already in the first switching cycle.

The non‐linear normalized nucleation rate distribution extracted from the fit of the data in Figure 4a by the SNNG model is shown in Figure 4b. Ferroelectric switching cycling results in a continuous shift of the non‐linear normalized nucleation rate distribution toward lower time values accompanied by an increase in the maximum of the distribution. This result suggests the continuous addition of M‐polar nucleation sites and the reduction of the energy required for nucleation while cycling. Figure 4c shows the evolution of both t_sw_ − τ_peak_ and ${\bar{t}}_{\mathrm{imp}}$ − τ_peak_ upon cycling. Interestingly, both t_sw_ − τ_peak_ and ${\bar{t}}_{\mathrm{imp}}$ − τ_peak_ are found to decrease linearly in the last 40 cycles. The continuous addition of M‐polar nucleation sites accompanied by lowering the energy required to form the nuclei would result in an increase of the nucleation contribution to the switching kinetics and, hence, in the reduction of t_sw_ − τ_peak_ while cycling. The reduction of ${\bar{t}}_{\mathrm{imp}}$ − τ_peak_ upon cycling is suggestive of the tendency toward the simultaneous occurrence of domain nucleation and wall motion of the already nucleated domains. In the studied case, electric field cycling does not alter the ${\bar{t}}_{\mathrm{imp}}$ > τ_peak_ relationship, thus a significant domain growth follows the nucleation in each cycle. Further insights into the kinetics of ferroelectric switching can be obtained by looking at the evolution of the single τ_peak_ and ${\bar{t}}_{\mathrm{imp}}$ during cycling (Figure 4d). Both τ_peak_ and ${\bar{t}}_{\mathrm{imp}}$ decrease upon cycling and follow a linear trend in the last 40 cycles. However, ${\bar{t}}_{\mathrm{imp}}$ decreases ten times faster than τ_peak_. The reduction of ${\bar{t}}_{\mathrm{imp}}$ can be related to an increase in domain wall velocity and/or the increase in the saturated density of nuclei of domains of opposite polarity, which results in more closely spaced domains.^[^ ^23^ ^]^ Ferroelectric switching in Al_0.85_Sc_0.15_N was proposed to generate bulk defects as a consequence of lateral domain wall motion.^[^ ^26^ ^]^ In agreement with this hot atom damage defect generation model, Zhu et al.^[^ ^40^ ^]^ noticed that domain wall motion in wurtzite‐type Al_1−x_B_x_N would involve bond breaking‐bond formation processes. Defects are known to act as pinning sites for domain wall motion, thus it is unlikely that the increasing defect concentration upon cycling would directly result in a faster domain wall motion.^[^ ^37^, ^38^, ^43^ ^]^ On the other hand, an increased disorder in the Al_0.85_Sc_0.15_N film due to defect generation can reduce the local energy barrier for polarization reversal, thus facilitating nucleation events.^[^ ^36^, ^37^ ^]^ A larger concentration of defects would then increase the number of domains and inevitably decrease the distance between them, causing ${\bar{t}}_{\mathrm{imp}}$ to get lower. It is worth also noticing that since the measured E_c_ reduces upon cycling, the ratio between the applied electric field and the measured E_c_ increases; hence, the domain wall motion gets faster. Nonetheless, the faster domain wall motion with cycling is only a direct consequence of the larger ratio between the applied electric field and the measured E_c_ and is not the root cause for the faster N‐ to M‐polar switching. Considering the absence of a clear wake‐up behavior for the studied capacitor structures unlike in the Al_1−x_B_x_N case discussed by Yazawa et al.^[^ ^23^, ^41^ ^]^, the analysis presented in this work shows that the increase in the number of M‐polar nucleation sites and the density of domain walls upon cycling is an intrinsic feature of wurtzite‐type AlN‐based ferroelectrics related to the progressive destabilization of the as‐grown and preferred N‐polarity.

## 3 Conclusion

Transient current integration measurements performed by a pulse switching method and domain imaging by PFM were used to investigate the kinetics of domain nucleation and wall motion during polarization reversal in Al_0.85_Sc_0.15_N capacitors. Under the application of electric field magnitudes below 5.0 MV cm^−1^, the switching kinetics is macroscopically represented by the KAI model, whereas for larger electric field magnitudes, the SNNG model provides a better fit of the experimental data due to the non‐negligible contribution of the non‐linear increase of the nucleation rate. For the electric field range used in this study (from 4.4 to 5.6 MV cm^−1^), domain wall movement significantly contributes to the switching kinetics. Time‐dependent visualization of the domain structure by stroboscopic PFM during N‐ to M‐polar transition confirms that ferroelectric switching proceeds through three main steps: i) nucleation of M‐polar domains; ii) sideways expansion of already nucleated M‐polar domains and simultaneous occurrence of additional nucleation events; iii) domain growth by lateral wall motion accompanied by a negligible contribution of nucleation. A non‐linear increase of both the domain nucleation rate and wall velocity is extracted from stroboscopic PFM data. The non‐linear evolution of the domain wall velocity during the switching process is currently not accounted for in any phenomenological model.

Electric field‐dependent evolution of the domain structure recorded by PFM showed that nucleation events occur in different regions according to which polarity between N‐ and M‐polar is nucleating, causing the domain pattern to depend on the polarization reversal direction. Transient current integration measurements are suggestive of a continuously faster N‐ to M‐polar switching while cycling due to the increasing number of M‐polar nucleation sites and density of domain walls, which could be correlated with an increased disorder in the Al_0.85_Sc_0.15_N film upon cycling.

The results of this work represent a clear step forward in the understanding of the complex domain structure dynamics involved in the ferroelectric switching process of wurtzite‐type Al_1−x_Sc_x_N, which is of the utmost importance for exploiting the full potential of this ferroelectric material in memory, power electronics, and innovative computing applications. By illustrating the reason for the progressive reduction of the electric field required to attain the N‐ to M‐polar ferroelectric switching in Al_1−x_Sc_x_N, the results of this study can be useful to improve the reliability and precisely control the multilevel update of the switched polarization in ferroelectric Al_1−x_Sc_x_N devices.

## 4 Experimental Section

### Sample Fabrication

The ferroelectric capacitor stack was deposited on a p‐type silicon (Si) substrate. First, 55 nm of Ti followed by 3 nm W were deposited as BE by direct current sputtering at room temperature. A 60 nm thick Al_0.85_Sc_0.15_N film was then deposited by radio frequency co‐sputtering from aluminum and scandium targets at 400 °C. A 25 nm thick TiN TE was deposited at room temperature. All the sputtering processes were performed in an ultra high‐vacuum sputter cluster from Bestec GmbH without breaking the vacuum condition between each deposition.

A lithographic step (Heidelberg Instruments µPG 101 laser lithography tool), followed by deposition of 5 nm Ti and 50 nm platinum (Pt) by electron‐beam evaporation (Bestec GmbH tool), lift‐off in acetone and ultrasonic bath, and inductively coupled plasma (ICP) etching (Plasmalab System133 from Oxford Instruments) were performed to structure the TE and improve the TE contact. Photoresist spinning and baking followed by ICP etching were performed to remove the Al_0.85_Sc_0.15_N from an edge of the sample and directly contact the BE with the probe needles. Finally, the photoresist was removed in acetone and ultrasonic bath. More detailed information on the relevant process parameters and structural characterization were previously published.^[^ ^26^ ^]^ The fabrication process described above refers to the capacitors used for the transient current integration measurements. PFM required thinner TE and contact layers to improve the spatial resolution limit for domain imaging.^[^ ^36^ ^]^ For this reason, the capacitor stack used for PFM was the same as the stack used for the transient current integration measurements, except for a thinner TiN TE (10 nm) and Ti (5 nm)/Pt (10 nm) contact films.

### Transient Current Integration Measurements

The capacitor structures tested during transient current integration measurements had an area of ≈1345 µm^2^. All measurements were performed at room temperature on a Cascade Microtech probe station using Keithley 4225 pulse measurement units controlled by a Keithley 4200A‐SCS parameter analyzer. All electrical signals were applied to the TE while the BE was grounded. A different capacitor per measurement sequence was tested to acquire the data shown in Figure 1b. For the data shown in Figure 1b, the extracted P_r_ was normalized by the P_r_ of the last data point of the sequence, which reached the expected value of 130 µC cm^−2^ as reported in previous work.^[^ ^26^ ^]^ All the measurement sequences shown in Figure 4a were run up to the pulse width of ≈1 ms to avoid early electrical breakdown in the measured capacitors. The normalized polarization value was computed with respect to the maximum extracted P_r_ of all measurement sequences to check for the occurrence of possible fatigue and/or wake‐up phenomena. The parameter analyzer was controlled by a custom LabVIEW software to automatically run each measurement sequence and control the elapsed time between two consecutive measurement points of each sequence (≈2.2 s).

### Piezoresponse Force Microscopy

The capacitor structures probed with PFM had an area of ≈113 µm^2^. PFM imaging was performed in the dual alternating current (AC) resonance tracking (DART) mode on a commercial atomic force microscopy system (MFP3D, Asylum Research) using Pt‐coated Si tips (NSC18/Pt, Mikromasch). The AC modulation voltage was 0.8 V in amplitude and ≈350 kHz in frequency. The high‐voltage switching pulses were generated via a high‐voltage amplifier module (Asylum Research) with a pulse rising time in the order of 0.1 ms. All voltages were applied to the TE through the tip, while the BE was grounded. The Pt‐coated Si tips used in this study were able to withstand the switching current of the tested capacitors. No correlation was observed by comparing the TE surface morphology with the PFM images acquired, thus confirming the authenticity of the reported domain configurations. All the PFM phase images in this study were acquired as a separate step compared to the voltage pulse application. This approach allowed us to avoid the occurrence of artifacts coming from large current densities during the switching process of the Al_0.85_Sc_0.15_N film. To get a time‐dependent evolution of the domain structure by PFM that can be compared with the results of the transient current integration measurements, a voltage pulse of 30 V in amplitude and 10 ms in width was applied to reset the capacitor in the N‐polar state after the acquisition of each PFM phase image in Figure 2a (and Figure S5, Supporting Information). In Figure 3, the PFM phase images were acquired after applying voltage pulses with a fixed width of 1 ms. The nucleation event was described as the emergence of new oppositely poled domains compared to the surrounding matrix manifested by the inverse phase contrast in PFM images. The average lateral domain wall velocity was estimated by monitoring the growth of several individual domains in Figure 2a before coalescence with the neighboring domains. The two‐dimensional nucleation density was estimated by counting the total number of nuclei over the imaged area. The nucleation rate was calculated by counting the number of new nuclei at a specific write pulse width over the effective N‐polar area.

### Mathematical Fitting

The mathematical fit of the experimental switching kinetics data was performed using the equations for the KAI (Equation S1, Supporting Information), NLS (Equation S4, Supporting Information), and SNNG (Equation S8, Supporting Information) models reported in Section S1 (Supporting Information). The best‐fitting function for each model was determined by minimizing the non‐linear least squares. For the KAI model, the parameter n was fixed to 2, and the best t_0_ was determined during the fit. For the NLS model, the fitting variables were A, w and t_1_. For the SNNG model ,the parameter m was fixed to 5 during the fit, whereas ν^2^ · N(∞) and α were used as fitting variables.

## Conflict of Interest

The authors declare no conflict of interest.

## Supporting information

Supporting Information

## Data Availability

The data that support the findings of this study are available from the corresponding author upon reasonable request.
