# Supplementary material for: Kinetics of N‐ to M‐Polar Switching in Ferroelectric Al1−xScxN Capacitors
Source: Adv Sci (Weinh). 2024 Feb 14;11(16):2308797. doi: 10.1002/advs.202308797 (PMC11040360; doi:10.1002/advs.202308797)
Supplement: Supplementary file 1 — Supporting Information [file ADVS-11-2308797-s001.pdf]

ADVANCED SCIENCE
Open Access

Supporting Information

for *Adv. Sci.*, DOI 10.1002/advs.202308797

Kinetics of N- to M-Polar Switching in Ferroelectric $Al_{1-x}Sc_xN$ Capacitors

Roberto Guido, Haidong Lu, Patrick D. Lomenzo, Thomas Mikolajick, Alexei Gruverman and Uwe Schroeder**

Supporting Information

Kinetics of N- to M-polar Switching in Ferroelectric $Al_{1-x}Sc_xN$ Capacitors

Roberto Guido, Haidong Lu, Patrick D. Lomenzo, Thomas Mikolajick, Alexei Gruverman, Uwe Schroeder**

Roberto Guido, Patrick D. Lomenzo, Thomas Mikolajick, Uwe Schroeder
NaMLab gGmbH, Noethnizer Strasse 64a, 01187 Dresden, Germany

Haidong Lu, Alexei Gruverman
Department of Physics and Astronomy, University of Nebraska, Lincoln, NE 68588, USA

Roberto Guido, Thomas Mikolajick
Chair of Nanoelectronics, Technische Universität Dresden, Noethnizer Strasse 64, 01187 Dresden, Germany

*E-mail: roberto.guido@mailbox.tu-dresden.de, Uwe.Schroeder@namlab.com

Section S1: Overview on ferroelectric switching models

Several phenomenological theories have been presented to model and predict the process of ferroelectric switching. In this section an overview on the three main models used to describe the switching kinetics in ferroelectrics is presented. All three models were considered to interpret the experimental data in this work; hence, this section aims to provide the basic theoretical background required to follow the discussion in the main manuscript.

In 1971, Ishibashi and Takagi applied the classical results by Kolmogorov and Avrami to ferroelectric switching.[1] The theory developed by Ishibashi and Takagi is commonly referred to as Kolmogorov-Avrami-Ishibashi (KAI) model. The KAI model was formulated considering an infinite ferroelectric crystal in which switching occurs by growth of nuclei through domain wall motion until impingement. From an application point of view of the theory, the assumption of an infinite crystal implies that the KAI model can be applied if the "width" of the ferroelectric film is much larger than the distance between the nuclei. It is worth mentioning that the KAI model assumes the domain wall velocity is constant under the application of a constant electric

field. The volume fraction of the ferroelectric f switched at the time t is expressed by Equation S1.[1]

$$f = 1 - e^{-(t/t_0)^n} \qquad (S1)$$

where t_0 is the characteristic time for polarization evolution, and n is the Avrami exponent. The t_0 depends on the domain wall velocity which increases with the magnitude of the applied electric field. When t_0 is smaller, the ferroelectric switching process is faster.

The Avrami exponent n equals to either the growth dimensionality D in the assumption of pre-existing nuclei, or to $D + 1$ assuming a constant nucleation rate throughout the switching period. A one-dimensional growth ($D = 1$) means that the domain wall of a plate-like nuclei moves along one direction. A two-dimensional growth ($D = 2$) corresponds to the growth of a cylindrical domain in two dimensions. A three-dimensional growth ($D = 3$) considers the growth of spherical nuclei in three dimensions. For thin films, $n = 2$ is often assumed because of the faster domain growth along the thickness of the ferroelectric film compared to the sideways domain wall motion.[2] These three cases are schematically illustrated in Figure S1.

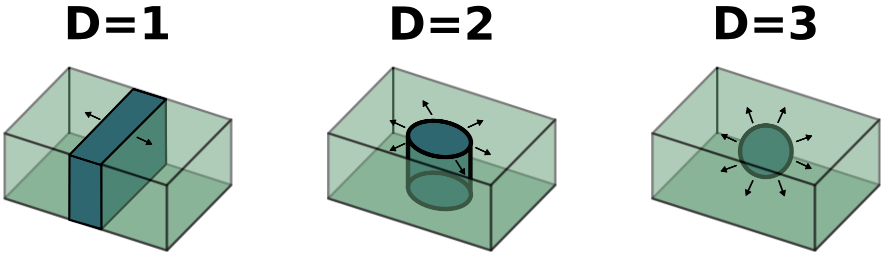

Figure S1. Schematic illustration of the shape of the ferroelectric domains according to the dimensionality of the growth, which can be one-dimensional ($D = 1$), two-dimensional ($D = 2$) or three-dimensional ($D = 3$). The arrows indicate the direction of growth of the ferroelectric domain.

A limitation of the KAI model is that physically different switching scenarios can result in the same evolution of the switched volume with time. For instance, the case of two-dimensional growth with pre-existing nuclei gives rise to an equivalent expression of f as the case of a one-dimensional growth accompanied by a constant nucleation rate throughout the switching period. Therefore, a direct observation of the switching process is required to fully capture how the polarization reversal occurs.

The KAI model describes the ferroelectric switching in single crystal materials well, but it was shown to not fully capture the kinetics of polarization reversal in polycrystalline ferroelectric thin films, such as in the case of lead zirconate titanate (PZT) or doped hafnia, over switching times more than 1-2 decades.[3–6] The KAI model does not produce the logarithmically slow increase of f with t observed for thin films.[3] Moreover, a variation of the electric field applied to the ferroelectric leads only to a horizontal shift along the time-axis and it does not account

for a change in slope of the f versus t curve typically observed in thin films.[3] Thus, Tagantsev et al.[3] proposed to model the switching kinetics in thin films by considering areas with independent nucleation dynamics. Hence, the switching kinetics is described in terms of the distribution function of the nucleation probabilities in these areas. Since the switching in each area is dominated by nucleation of reversed domains and domain growth does not significantly influence the switching kinetics, the model is referred to as nucleation-limited-switching (NLS). In the NLS model, the volume fraction of the ferroelectric f switched at the time t is expressed by Equation S2.[4]

$$f = \int_{-\infty}^{+\infty}\left[1 - e^{-(t/t_0)^n}\right] \cdot F(\log t_0)\, d(\log t_0) \qquad \text{(S2)}$$

where $F(\log t_0)$ is the distribution function of the characteristic switching time t_0, and n is the effective dimension of domain growth (also in this case $n = 2$ is usually assumed for thin films). A Lorentzian distribution of the local switching times can be assumed due to local electric field variations caused by dipole defects at domain pinning sites, and it is reported in Equation S3.[4]

$$F(\log t_0) = \frac{A}{\pi} \cdot \frac{w}{(\log t_0 - \log t_1)^2 + w^2} \qquad \text{(S3)}$$

where A is a normalization constant, w is the half-width at half-maximum of the distribution function, and $\log t_1$ is the center of the distribution function.

Considering that a double exponential function $e^{-\left(10^{\log t}/10^{\log t_0}\right)^n}$ for $n > 1$ can be approximated as a step function centered in $\log t_0 = \log t$, Equation S2 can be simplified as shown in Equation S4.[4]

$$f = \frac{A}{\pi} \cdot \left[\arctan\left(\frac{\log t - \log t_1}{w}\right) + \pi/2\right] \text{(S4)}$$

Increasing the applied electric field magnitude, the distribution of the local switching times shifts toward lower time scales as well as it gets narrower, thus causing the curve described in Equation S4 to become more abrupt.

Recently, Yazawa et al.[7] proposed an extension of the classical KAI model to describe the anomalously abrupt switching in wurtzite-type ferroelectrics under the application of relatively large electric fields magnitudes. This model considers the case of a non-linear increase of the nucleation rate which occurs simultaneously with substantial domain growth and impingement, thus it is referred to as simultaneous non-linear nucleation and growth (SNNG) model. Under the assumption of the ferroelectric as an infinite system in which the domain growth ends with impingement of neighbouring domains, the volume fraction of the ferroelectric f switched at the time t is expressed by Equation S5.

$$f = 1 - e^{-\int_0^t \dot{N}(\tau)\cdot V(t,\tau)\, d\tau} \qquad \text{(S5)}$$

where $\dot{N}$ is the nucleation rate, V is the volume of each reversed domain, and τ is the nucleation time. The mth order function in Equation S6 is used to account for the non-linear increase in the nucleation rate $\dot{N}$.

$$\dot{N}(\tau) = \alpha \cdot N(\infty) \cdot m \cdot \tau^{m-1}\, e^{-\alpha\cdot\tau^{m}} \quad \text{(S6)}$$

where α is a constant determining the position of the maximum of the nucleation rate, $N(\infty)$ is the saturated density of nuclei of oppositely poled domains. Under the assumption of isotropic two-dimensional domain growth, the volume of each nucleus of reversed domain can be written as reported in Equation S7.

$$V(t,\tau) = 2\pi \cdot d \cdot \nu^2 \cdot (t-\tau)^2 \qquad \text{(S7)}$$

Where d is the film thickness and ν is the domain wall velocity. By substituting the nucleation rate distribution in Equation S6 and the expression for the volume of each nucleus of reversed domain in Equation S7 into Equation S5, the volume fraction of the ferroelectric f switched at the time t can be expressed as reported in Equation S8.

$$f = 1 - e^{-2\pi\cdot d\cdot\nu^2\cdot N(\infty)\cdot\alpha\cdot m\cdot\int_0^t \tau^{m-1}\cdot(t-\tau)^2\cdot(e^{-\alpha\tau^m})d\tau} \qquad \text{(S8)}$$

From fitting the experimental switching kinetics data with the function reported in Equation S8, the time at which the peak of nucleation rate occurs τ_{peak} and the average time between impingement of neighbouring reversed domains $\bar{t}_{imp}$ can be calculated using the expression in Equation S9 and S10, respectively.

$$\tau_{peak} = \sqrt[m]{\frac{m-1}{m\cdot\alpha}} \qquad \text{(S9)}$$

$$\bar{t}_{imp} = \frac{1}{2\nu\cdot\sqrt{dN(\infty)}} \qquad \text{(S10)}$$

It is worth mentioning that the expression of the $\bar{t}_{imp}$ in Equation S10 is calculated assuming a two-dimensional domain growth and uniformly distributed nuclei of reversed domains. The first assumption allows one to express the saturated two-dimensional nucleation density of oppositely poled domains ($N^{2D}(\infty)$) as in Equation S11, and the second assumption implies that the average distance of a domain to its nearest neighbor ($\bar{l}_{nearest}$) can be expressed as in Equation S12. Thus $\bar{t}_{imp}$ is computed by dividing $\bar{l}_{nearest}$ by two times the domain wall velocity.

$$N^{2D}(\infty) = dN(\infty) \qquad \text{(S11)}$$

$$\bar{l}_{nearest} = \frac{1}{\sqrt{N^{2D}(\infty)}} \qquad \text{(S12)}$$

Being an extension of the KAI model, the SNNG collapses to KAI under certain conditions. In particular, the SNNG model collapses to KAI when $\tau_{peak} < \bar{t}_{imp}$ because the peak of

nucleation rate precedes the significant domain growth. The extended model proposed by Yazawa et al.[7] covers then both the case of significant domain growth and impingement after nucleation ($\tau_{peak} < \bar{t}_{imp}$) and the regime where domain growth and increase of the nucleation rate occur simultaneously ($\tau_{peak} > \bar{t}_{imp}$). The latter case corresponds to a larger activation field for domain growth compared to nucleation. The three models described in this section are schematically illustrated in Figure S2.

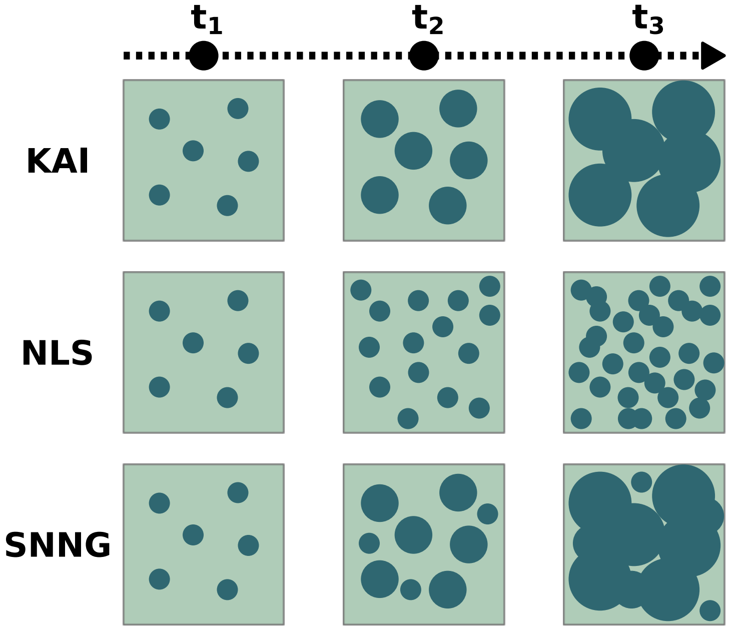

Figure S2. Schematic illustration of how the ferroelectric switching process proceeds in time for the KAI (in the case of domain growth from pre-existing nuclei of oppositely poled domains), NLS and SNNG phenomenological models. The oppositely poled domains appearing and/or growing during the switching process are indicated in dark green. The domain pattern is schematically illustrated for three instants of time (t_1, t_2 and t_3) satisfying the relationship $t_1 < t_2 < t_3$.

Section S2: Phenomenological model fit of the data acquired by transient current integration measurements

Three phenomenological models (KAI, NLS and SNNG) were considered in the proposed analysis to fit the experimental data obtained by transient current integration measurements. The aim of this section is to provide additional information regarding the fit of the experimental data. Figure S3 shows the fit of the normalized polarization versus pulse width for three different electric field magnitudes (4.4, 5.0 and 5.6 MV cm^{-1}) by using the KAI, NLS and SNNG models, as well as the normalized non-linear nucleation rate distribution for each electric field magnitude when the SNNG model is applied to fit the data. When the applied electric field magnitude is 4.4 MV cm^{-1} the KAI and SNNG models provide a better fit to the switching kinetics compared to the NLS. The solutions obtained using the KAI and SNNG models overlap

each other. This result agrees with the fact that the SNNG model is an extension of the KAI model, with which the SNNG produces the same result if significant domain growth follows the peak in nucleation rate, as in the present case.[7] Increasing the applied electric field magnitude to 5.0 MV cm^{-1}, a discrepancy between the KAI and SNNG model starts to emerge with the latter that better fits the data. In particular, the larger the electric field magnitude that is applied to the capacitor, the more abrupt the ferroelectric switching process becomes; thus, increasing the discrepancy between the KAI and SNNG models. When 5.6 MV cm^{-1} are applied, the KAI model clearly does not fit adequately the data in Figure S3c, whereas a good agreement with the SNNG model is found. For both 5.0 MV cm^{-1} and 5.6 MV cm^{-1}, the fit provided by the NLS model does not accurately follow the normalized polarization evolution with pulse width as well as with using the SNNG model. The reason for the progressively increasing difference between the solutions provided by the KAI and SNNG models with increasing applied electric field magnitude is related to the contribution to the switching kinetics of the non-linear increase of the nucleation rate. As discussed in the main text, increasing the applied electric field magnitude, the switching process becomes more and more abrupt due to the larger contribution to the switching kinetics of the non-linear increase of the nucleation rate. Indeed, by comparing Figure S3d, e and f, it is observable that the magnitude of the normalized nucleation rate values increases and the nucleation rate peak gets closer to the slope of the normalized polarization versus pulse width curve, with increasing applied electric field.

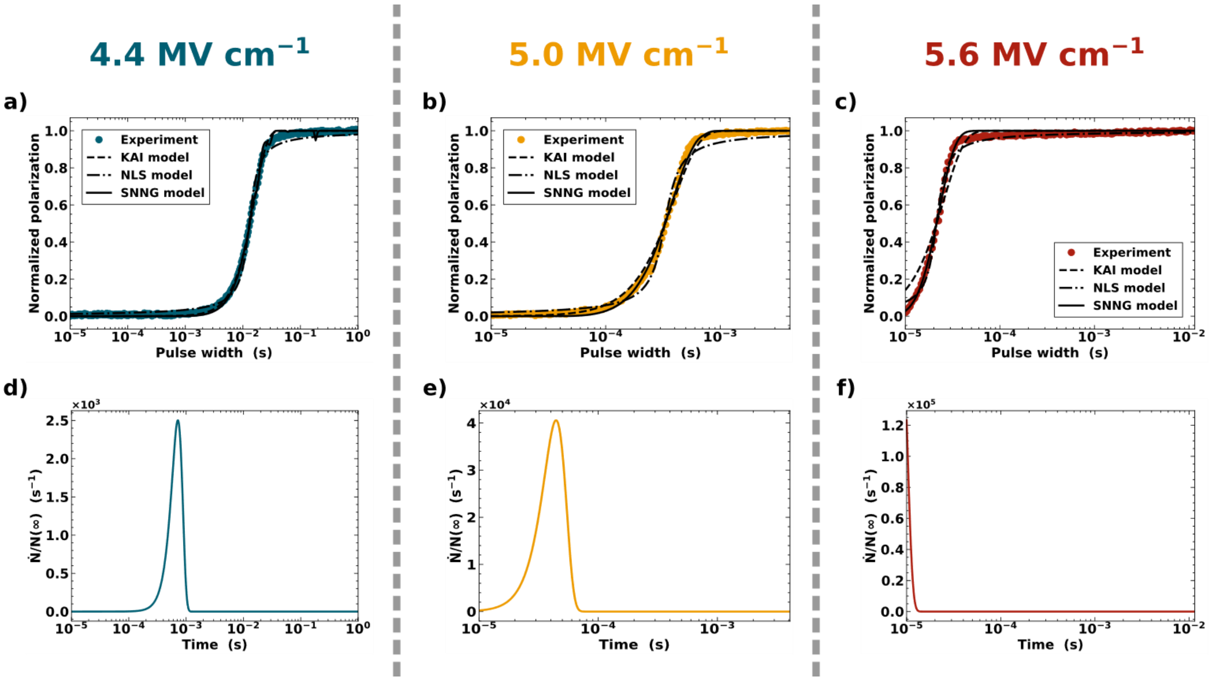

Figure S3. Normalized polarization extracted from transient current integration measurements vs. pulse width for applied electric field magnitudes of (a) 4.4, (b) 5.0 and (c) 5.6 MV cm^{-1}. The best fitting function is shown for the KAI, NLS and SNNG models. The normalized non-linear

nucleation rate distribution ($\dot{N}/N(\infty)$) for the SNNG model is reported for applied electric field magnitudes of (d) 4.4, (e) 5.0 and (f) 5.6 MV cm^{-1}.

In the main text, the crossover between KAI and SNNG models with applied electric field magnitude is discussed in terms of difference between the average impingement time ($\bar{t}_{imp}$) or switching time (t_{sw}) and the time at which the non-linear increase of the nucleation rate peaks (τ_{peak}). Table S1 reports standard deviation of the characteristic switching time (t_0) for the applied electric field magnitudes that resulted in normalized polarization versus pulse width fit with the KAI model, and of $\bar{t}_{imp} - \tau_{peak}$ and $t_{sw} - \tau_{peak}$ when the SNNG model was used to fit the data.

Table S1. Standard deviation of the characteristic switching time for the KAI model (σ_{t_0}) and of the difference between the average impingement time or switching time and the time at which the non-linear increase of the nucleation rate peaks (respectively $\sigma_{\bar{t}_{imp}-\tau_{peak}}$ and $\sigma_{t_{sw}-\tau_{peak}}$) for the SNNG model. The standard deviations are listed according to the applied electric field magnitude of the write pulse applied during the transient current integration measurements.

E (MV cm^{-1})	σ_{t_0} (μs)	$\sigma_{\bar{t}_{imp}-\tau_{peak}}$ (μs)	$\sigma_{t_{sw}-\tau_{peak}}$ (μs)
4.4	54.70	---	---
4.6	17.13	---	---
4.8	5.06	---	---
5.0	---	5.55	2.89
5.2	---	1.03	0.18
5.4	---	0.89	0.49
5.6	---	0.44	0.25

Since the SNNG model produces the same result of the KAI model if significant domain growth follows the peak in nucleation rate, the SNNG model can be used to fit the data acquired by transient current integration measurements also for the applied electric field magnitudes below 5.0 MV cm^{-1}. Using the equation reported in Section S1, the domain wall velocity (ν) can be plotted versus the saturated two-dimensional nucleation density of oppositely poled domains ($N^{2D}(\infty)$). The result is reported in Figure S4 and it shows that the domain wall movement gets faster by increasing the applied electric field magnitude.

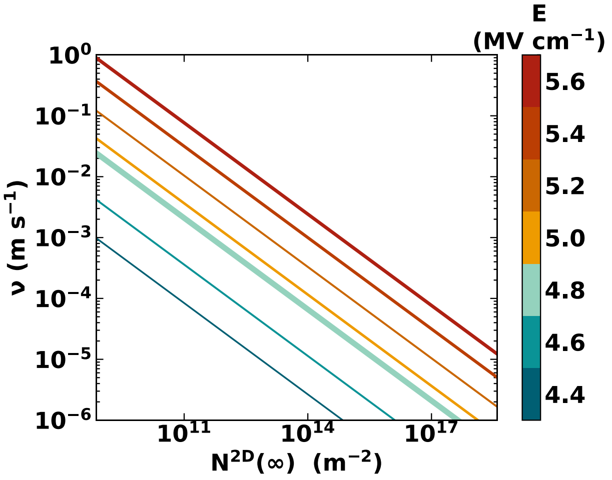

Figure S4. Domain wall velocity (ν) vs. saturated two-dimensional nucleation density of oppositely poled domains ($N^{2D}(\infty)$) extracted from the fit using the SNNG model of the data acquired by transient current integration measurements for each applied electric field magnitude (E) between 4.4 and 5.6 MV cm^{-1}. The shaded region indicates the confidence interval considering three standard deviations of the fitting parameters of the SNNG model. The $N^{2D}(\infty)$ axis is limited to a range from one nucleus in the measured capacitor to one nucleus per unit cell.

Section S3: Calculation of the depolarization field

In ferroelectric thin films, a depolarization field directed in the opposite direction with respect to the dipole moment originates from the incomplete compensation of the polarization charge at the interface with the electrodes and raises significant reliability concerns for storage applications, especially in terms of retention of the memory state.[8–10] Considering an ideal one-dimensional case of a perfectly insulating ferroelectric film and neglecting the presence of interface trap states at the metallic electrode interface, the electric field in the ferroelectric at the equilibrium condition can be computed starting from the Poisson's equation under appropriate boundary conditions.[8] The mathematical expression of the magnitude of the electric field in the ferroelectric (E_{ferro}) is reported in Equation S13.

$$E_{ferro} = \frac{\sigma - P}{\varepsilon_0 \cdot \varepsilon_{ferro}} \qquad (S13)$$

Where P is the polarization in the ferroelectric film, σ is the area density of the compensation charges induced in the metal electrodes, ε_0 is the vacuum permittivity and ε_{ferro} is the static dielectric constant of the ferroelectric material. Equation S13 contains two contributions: the depolarization field arising from the polarization charges and the compensation from charges induced in the metal electrodes. Thus, in the absence of screening charges, the magnitude of the depolarization field (E_{dep}) can be written as in Equation S14.

$$E_{dep} = -\frac{P}{\varepsilon_0 \cdot \varepsilon_{ferro}} \qquad (S14)$$

For the case of the presented study, the remanent P in the ferroelectric film is ≈130 μC cm^{-2} as reported in a previous work.[11] Considering the scandium content of the ferroelectric film, the $\varepsilon_{\mathrm{ferro}}$ is expected to be ≈11.5.[12] Thus, the E_{dep} is estimated as ≈127.7 MV cm^{-1}.

It is worth noticing that the expression of E_{dep} in Equation S14 is actually a simplified treatment of the depolarization field and that in real scenarios E_{dep} is influenced by other effects, such as the finite electronic screening length of the electrode and the presence of “dead layers” at the interface between the ferroelectric film and the electrode.[8,13] Nonetheless, the definition of E_{dep} as in Equation S14 is suitable for supporting the hypothesis in the main manuscript that the activation field computed by Merz’s empirical expression corresponds to the threshold for the transition of domain wall motion from creep regime to flow regime. Considering pinning sites as dipoles with fixed polarity to which neighboring dipoles tend to align and that domain wall flow implies the switching of the pinned region, Zhao et al.[14] noted that the average electric field to overcome at the pinning site for the onset of domain wall flow motion is the E_{dep} computed as in Equation S14.

Section S4: Suitability of the NLS model to fit the evolution in time of the switched volume fraction extracted from stroboscopic PFM data

The fit of the data acquired by transient current integration measurements showed that below 5.0 MV cm^{-1}, the switching kinetics in ferroelectric $Al_{0.85}Sc_{0.15}N$ capacitors is described macroscopically by the KAI model. In contrast, the SNNG model better fits the experimental data for larger applied electric field magnitudes. However, for the applied electric field magnitude of 5.0 MV cm^{-1}, the switched volume fraction extracted by the stroboscopic PFM experiment and plotted versus the pulse width (Figure 2b of the main manuscript) can be equally well described mathematically by all the three phenomenological models considered in this work (KAI, NLS and SNNG models). When phenomenological models are applied to explain experimental data, it is crucial to understand if the model fit has physical meaning or is pure mathematics. Additional stroboscopic PFM data acquired with a write pulse amplitude of 4.5 MV cm^{-1} are discussed here to discriminate whether the NLS model description of the time-dependent switched volume fraction extracted from PFM has physical meaning. The time-dependent PFM phase images are shown in Figure S5.

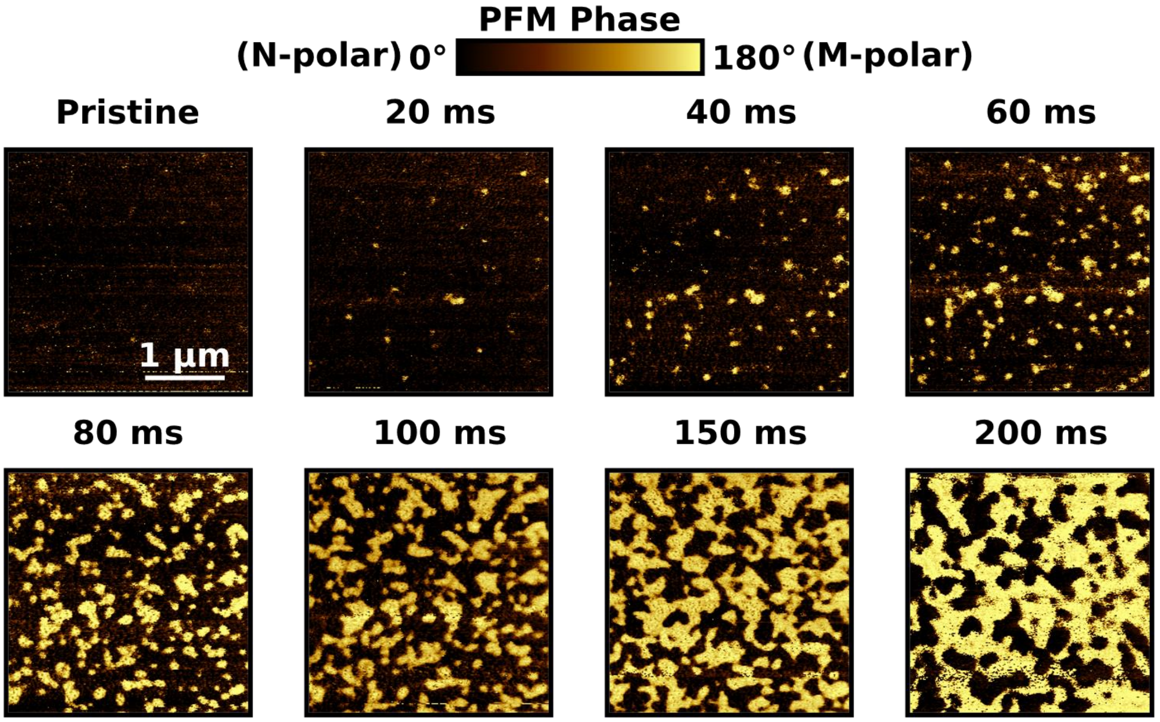

Figure S5. PFM phase images (3 µm x 3 µm) of the time-dependent switching behavior when applying a 4.5 MV cm^{-1} electric field directed toward the TE of an initially pristine capacitor. The scale bar of 1 µm applies to all PFM phase images.

In Figure S6a, the switched volume fraction extracted from the PFM phase images in Figure S5 is plotted versus the applied write pulse width with the mathematical fit of the data using the KAI, NLS and SNNG models. Similar to the fit of the data presented in Figure 2b of the main manuscript, looking only at the data points in Figure S6a, it is difficult to conclude which phenomenological model better explains the experimental data. Scanning probe tip degradation for longer pulse widths than 200 ms prevented acquiring additional data points. However, the suitability of the NLS model to describe the switching kinetics in ferroelectric $Al_{0.85}Sc_{0.15}N$ capacitors can be discussed considering the fit of the data in Figure 2b of the main manuscript and Figure S5a. As mentioned in Section S1, a decrease in the applied electric field magnitude for the NLS model causes a shift of the Lorentzian distribution of the local switching times toward longer time scales and its broadening. Both the shift and the change in width of the Lorentzian distribution function with applied electric field must be observed to validate the application of the NLS model for explaining the experimental data. Figure S6b reports the Lorentzian distribution function of the local switching times obtained from the NLS model fit of the switched volume fraction extracted from the PFM phase images for the two electric field magnitudes of 5.0 and 4.5 MV cm^{-1}. As expected, decreasing the applied electric field magnitude from 5.0 to 4.5 MV cm^{-1} results in a shift of the center of the Lorentzian distribution toward longer time scales. Nonetheless, the half-width at half-maximum of the Lorentzian

distribution function does not increase with the decrease in applied electric field magnitude. The absence of an increase in the half-width at half-maximum of the Lorentzian distribution of the local switching times for the NLS model when the applied electric field magnitude is decreased from 5.0 to 4.5 MV cm^{-1} supports the explanation of the switching kinetics in $Al_{0.85}Sc_{0.15}N$ capacitors provided by KAI and SNNG models even for the data acquired through stroboscopic PFM while weakening the suitability of the solution provided by the NLS model.

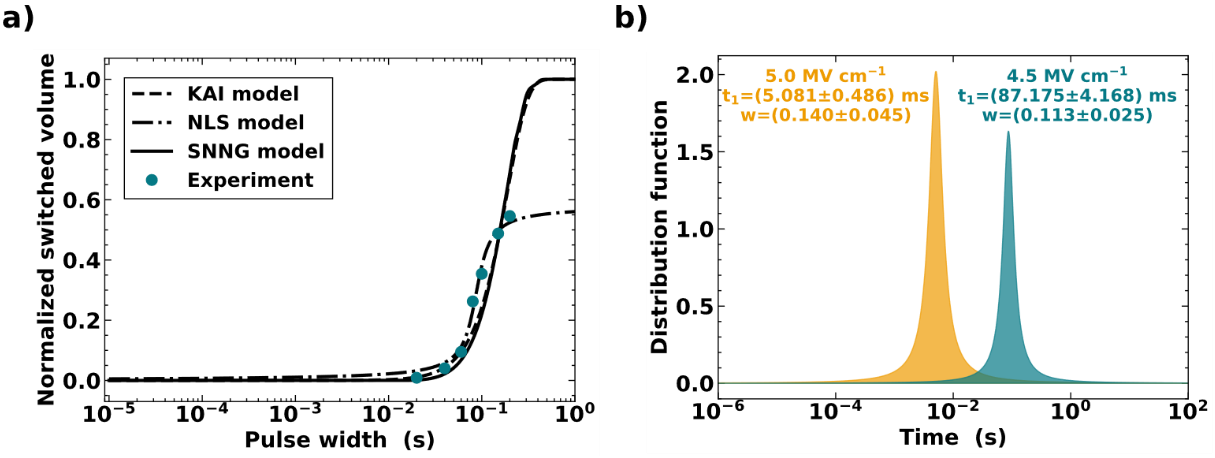

Figure S6. (a) Fitting of the normalized volume fraction of switched domains extracted from the PFM phase images in Figure S5 vs. pulse width by KAI, NLS and SNNG models. (b) Lorentzian distribution of the local switching times for the NLS model fit of the switched volume fraction extracted from the PFM phase images for the two electric field magnitudes of 5.0 and 4.5 MV cm^{-1}. The center (t_1) of the Lorentzian distribution and the half-width at half-maximum (w) values are reported in the figure for each distribution.

Section S5: Extraction of the reference coercive field value

A transient current measurement was performed on a ferroelectric capacitor initially in the pristine state to extract the magnitude of the coercive field for the N- to M-polar switching. The result is shown in Figure S7. The electric field magnitudes associated with the peaks in transient current were used to derive the magnitude of the positive (E_{c+}) and negative (E_{c-}) coercive fields: $E_{c+} \approx 5.0$ MV cm^{-1} and $E_{c-} \approx 6.3$ MV cm^{-1}. Thus, the reference coercive field magnitude for the N- to M-polar transition used in the main manuscript is 6.3 MV cm^{-1}.

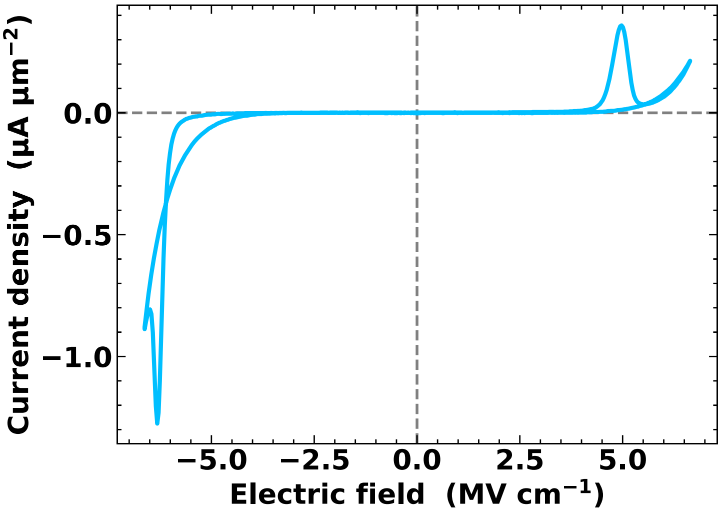

Figure S7. Transient current vs. applied electric field measured by applying first a negative and then a positive voltage on the TE of a pristine ferroelectric capacitor. The BE was grounded during the measurement. The measurement frequency was 2.5 kHz.

Section S6: On the origin of the faster ferroelectric switching by transient current integration measurements compared to the stroboscopic PFM experiment

In the main text, experimental data acquired by both transient current integration measurements and stroboscopic PFM at a fixed electric field magnitude of 5.0 MV cm^{-1} show that ferroelectric switching from N- to M-polar in $Al_{0.85}Sc_{0.15}N$ can be described using the SNNG model. However, even though the magnitude of the applied electric field during the write pulse is the same for both transient current integration measurements and stroboscopic PFM, the polarization reversal is faster during the transient current integration measurements compared to the time-dependent PFM experiment. A difference between the two types of measurements is the pulse sequence. Only one positive voltage pulse to reset the ferroelectric capacitor in the N-polar state is used for the time-dependent PFM experiment, whereas two must be applied for the transient current integration measurements for compensating for the capacitive displacement and leakage current components. Moreover, the starting pulse width of the pulse sequence is different between the two experiments (see Figure 1b and Figure 2b in the main manuscript). To understand if the starting pulse width of the measurement sequence had an impact on the evolution of the normalized polarization versus pulse width, the following experiment was performed. The measurement sequence (at 5.0 MV cm^{-1} for the applied electric field magnitude during the write pulse) was applied to six different initially pristine ferroelectric capacitors starting from a different pulse width per capacitor between 10 µs and ≈1 ms. The normalized polarization versus pulse width for the six pulse width values investigated is reported in Figure S8a. A dependence of the switching kinetics in the $Al_{0.85}Sc_{0.15}N$ on the starting pulse width of the measurement sequence emerges from Figure S8a. By applying longer

pulses from the beginning of the measurement sequence, the ferroelectric switching process becomes more abrupt, as shown by plotting the slope of the transition part of the curves in Figure S8a with respect to the starting pulse width of the measurement sequence (inset in Figure S8a). Moreover, the starting pulse width of the measurement sequence influences the normalized polarization value achievable by applying the same write pulse width. For instance, even though the normalized polarization saturates at 1 ms for the case of starting pulse width of 10 µs, applying directly a 1 ms long pulse as first one results in only ≈20% of the maximum polarization. This observation is suggestive of the dependence of the switched polarization in $Al_{0.85}Sc_{0.15}N$ on the time under electrical stress. To verify this hypothesis the data reported in Figure S8a were plotted by considering the cumulative normalized polarization as result of the cumulative time under electrical stress, which was obtained from summing up the pulse widths of consecutive write pulses (Figure S8b). The overlap of the cumulative normalized polarization data points in Figure S8b shows that N- to M-polar ferroelectric switching in $Al_{0.85}Sc_{0.15}N$ is the result of the accumulated electrical stimuli. The natural property of ferroelectrics to accumulate electrical excitation was already reported in the case of doped hafnia and was attributed to the progressive nucleation of the ferroelectric domains.[15] Nonetheless, differently from the work of Mulaosmanovic et al.[15], two positive voltage pulses which set back the capacitor to the N-polar state are applied after each write pulse. Thus, according to the discussion in the main manuscript, the observed cumulative N- to M-polar switching effect is suggestive of the fact that the time under an applied electric field directed toward the capacitor TE can increase the number of M-polar nucleation sites and density of domain walls, hence facilitating the subsequent N- to M-polar switching. However, a discrepancy between the measurement sequence starting at 10 µs and all the others can be observed in Figure S8b. This discrepancy can be explained by considering that the switching process is influenced by the time under electrical stress as well as other effects. In particular, the measurement sequence starting at 10 µs consists of much more applied pulses compared to all the other sequences. A larger number of applied pulses can influence the internal bias field and/or the electric field required to attain the N- to M-polar transition, thus impacting the normalized polarization value.

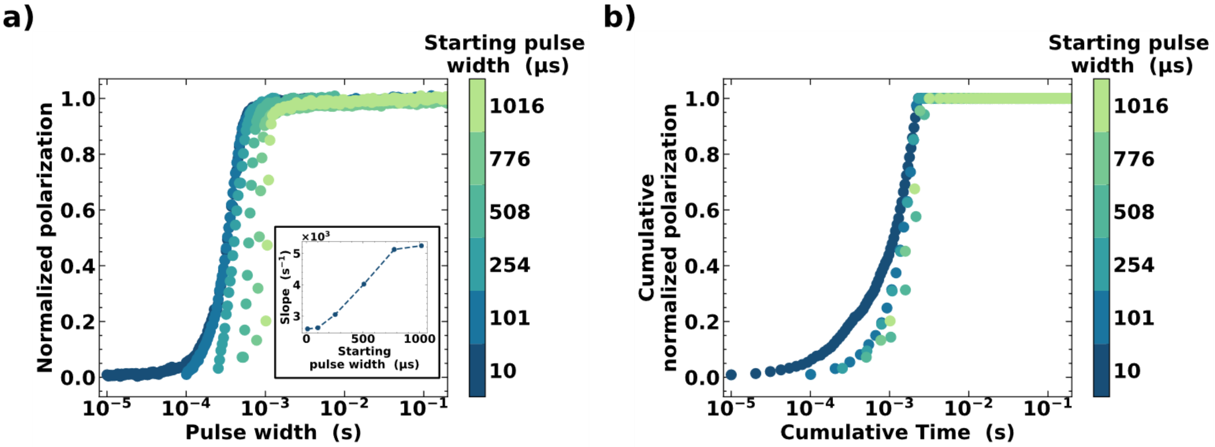

Figure S8. (a) Normalized polarization extracted from transient current integration measurements vs. pulse width for starting pulse width of the measurement sequence between 10 µs and ≈1 ms. The inset shows the slope of the normalized polarization vs. pulse width curve plotted with respect to the starting pulse width of the measurement sequence. (b) Cumulative normalized polarization extracted from transient current integration measurements vs. cumulative time of the applied write pulses for starting pulse width of the measurement sequence between 10 µs and ≈1 ms. For all measurement sequences, the applied electric field magnitude for the write pulse was fixed to 5.0 MV cm^{-1}.

The results reported in this section show that the faster polarization reversal during the transient current integration measurements compared to the time-dependent PFM experiment has to be in part ascribed to the pulse sequence characteristics, such as starting pulse width and number of pulses. Nonetheless, when a pulse with 1 ms width is applied to a pristine ferroelectric capacitor during stroboscopic PFM only ≈1% of the polarization is reversed whereas ≈20% of switched polarization is reported in Figure S8a. This still significant difference could be due to polarization relaxation and/or depolarization effects taking place in between the pulse application and the PFM images acquisition (which is much longer compared to the time for applying the reading pulses during transient current integration measurements), as well as to area-dependent effects (capacitors measured by PFM had an area of ≈113 μm^2 whereas the area of the capacitors measured by transient current integration measurements was ≈1345 μm^2). Further study is required to grasp all the details of the complex mechanisms involved in ferroelectric switching of wurtzite-type $Al_{1-x}Sc_xN$.

References

(1) Ishibashi, Y.; Takagi, Y. Note on Ferroelectric Domain Switching. *J. Phys. Soc. Jpn.* **1971**, *31* (2), 506–510. https://doi.org/10.1143/JPSJ.31.506.
(2) Merz, W. J. Domain Formation and Domain Wall Motions in Ferroelectric $BaTiO_3$ Single Crystals. *Phys. Rev.* **1954**, *95* (3), 690–698. https://doi.org/10.1103/PhysRev.95.690.
(3) Tagantsev, A. K.; Stolichnov, I.; Setter, N.; Cross, J. S.; Tsukada, M. Non-Kolmogorov-Avrami Switching Kinetics in Ferroelectric Thin Films. *Phys. Rev. B* **2002**, *66* (21), 214109. https://doi.org/10.1103/PhysRevB.66.214109.

(4) Jo, J. Y.; Han, H. S.; Yoon, J.-G.; Song, T. K.; Kim, S.-H.; Noh, T. W. Domain Switching Kinetics in Disordered Ferroelectric Thin Films. *Phys. Rev. Lett.* **2007**, *99* (26), 267602. https://doi.org/10.1103/PhysRevLett.99.267602.

(5) Lohse, O.; Grossmann, M.; Boettger, U.; Bolten, D.; Waser, R. Relaxation Mechanism of Ferroelectric Switching in $Pb(Zr,Ti)O_3$ Thin Films. *Journal of Applied Physics* **2001**, *89* (4), 2332–2336. https://doi.org/10.1063/1.1331341.

(6) Buragohain, P.; Richter, C.; Schenk, T.; Lu, H.; Mikolajick, T.; Schroeder, U.; Gruverman, A. Nanoscopic Studies of Domain Structure Dynamics in Ferroelectric $La:HfO_2$ Capacitors. *Applied Physics Letters* **2018**, *112* (22), 222901. https://doi.org/10.1063/1.5030562.

(7) Yazawa, K.; Hayden, J.; Maria, J.-P.; Zhu, W.; Trolier-McKinstry, S.; Zakutayev, A.; Brennecka, G. L. Anomalously Abrupt Switching of Wurtzite-Structured Ferroelectrics: Simultaneous Non-Linear Nucleation and Growth Model. *Mater. Horiz.* **2023**. https://doi.org/10.1039/D3MH00365E.

(8) Mehta, R. R.; Silverman, B. D.; Jacobs, J. T. Depolarization Fields in Thin Ferroelectric Films. *Journal of Applied Physics* **2003**, *44* (8), 3379–3385. https://doi.org/10.1063/1.1662770.

(9) Wurfel, P.; Batra, I. P.; Jacobs, J. T. Polarization Instability in Thin Ferroelectric Films. *Phys. Rev. Lett.* **1973**, *30* (24), 1218–1221. https://doi.org/10.1103/PhysRevLett.30.1218.

(10) Black, C. T.; Farrell, C.; Licata, T. J. Suppression of Ferroelectric Polarization by an Adjustable Depolarization Field. *Applied Physics Letters* **1997**, *71* (14), 2041–2043. https://doi.org/10.1063/1.119781.

(11) Guido, R.; Mikolajick, T.; Schroeder, U.; Lomenzo, P. D. Role of Defects in the Breakdown Phenomenon of $Al_{1-x}Sc_xN$: From Ferroelectric to Filamentary Resistive Switching. *Nano Lett.* **2023**, *23* (15), 7213–7220. https://doi.org/10.1021/acs.nanolett.3c02351.

(12) Akiyama, M.; Umeda, K.; Honda, A.; Nagase, T. Influence of Scandium Concentration on Power Generation Figure of Merit of Scandium Aluminum Nitride Thin Films. *Applied Physics Letters* **2013**, *102* (2), 021915. https://doi.org/10.1063/1.4788728.

(13) Stengel, M.; Spaldin, N. A. Origin of the Dielectric Dead Layer in Nanoscale Capacitors. *Nature* **2006**, *443* (7112), 679–682. https://doi.org/10.1038/nature05148.

(14) Zhao, D.; Lenz, T.; Gelinck, G. H.; Groen, P.; Damjanovic, D.; de Leeuw, D. M.; Katsouras, I. Depolarization of Multidomain Ferroelectric Materials. *Nat Commun* **2019**, *10* (1), 2547. https://doi.org/10.1038/s41467-019-10530-4.

(15) Mulaosmanovic, H.; Mikolajick, T.; Slesazeck, S. Accumulative Polarization Reversal in Nanoscale Ferroelectric Transistors. *ACS Appl. Mater. Interfaces* **2018**, *10* (28), 23997–24002. https://doi.org/10.1021/acsami.8b08967.
